# Are Filter-Tipped Cigarettes Still Less Harmful than Non-Filter Cigarettes?—A Laser Spectrometric Particulate Matter Analysis from the Non-Smokers Point of View

**DOI:** 10.3390/ijerph13040429

**Published:** 2016-04-16

**Authors:** Maria Schulz, Alexander Gerber, David A. Groneberg

**Affiliations:** Institute of Occupational Medicine, Social Medicine and Environmental Medicine, Goethe-University, Theodor-Stern-Kai 7, Haus 9b, Frankfurt am Main 60590, Germany; gerber@med.uni-frankfurt.de (A.G.); groneberg@med.uni-frankfurt.de (D.A.G.)

**Keywords:** particulate matter, cigarette smoke, tobacco taxation, Automatic Environmental Tobacco Smoke Emitter

## Abstract

*Background:* Environmental tobacco smoke (ETS) is associated with human morbidity and mortality, particularly chronic obstructive pulmonary disease (COPD and lung cancer. Although direct DNA-damage is a leading pathomechanism in active smokers, passive smoking is enough to induce bronchial asthma, especially in children. Particulate matter (PM) demonstrably plays an important role in this ETS-associated human morbidity, constituting a surrogate parameter for ETS exposure. *Methods:* Using an Automatic Environmental Tobacco Smoke Emitter (AETSE) and an in-house developed, non-standard smoking regime, we tried to imitate the smoking process of human smokers to demonstrate the significance of passive smoking. Mean concentration (C_mean_) and area under the curve (AUC) of particulate matter (PM_2.5_) emitted by 3R4F reference cigarettes and the popular filter-tipped and non-filter brand cigarettes “Roth-Händle” were measured and compared. The cigarettes were not conditioned prior to smoking. The measurements were tested for Gaussian distribution and significant differences. *Results:* C_mean_ PM_2.5_ of the 3R4F reference cigarette: 3911 µg/m^3^; of the filter-tipped Roth-Händle: 3831 µg/m^3^; and of the non-filter Roth-Händle: 2053 µg/m^3^. AUC PM_2.5_ of the 3R4F reference cigarette: 1,647,006 µg/m^3^·s; of the filter-tipped Roth-Händle: 1,608,000 µg/m^3^·s; and of the non-filter Roth-Händle: 858,891 µg/m^3^·s. *Conclusion:* The filter-tipped cigarettes (the 3R4F reference cigarette and filter-tipped Roth-Händle) emitted significantly more PM_2.5_ than the non-filter Roth-Händle. Considering the harmful potential of PM, our findings note that the filter-tipped cigarettes are not a less harmful alternative for passive smokers. Tobacco taxation should be reconsidered and non-smoking legislation enforced.

## 1. Introduction

Tobacco smoke is a major environmental pollutant that endangers human life from the unborn to the elderly [1,2,3]. Environmental tobacco smoke (ETS) consists of mainstream smoke (MSS; 15%), which is exhaled by the smoker, and side-stream smoke (SSS; 85%), which is emitted from a smoldering cigarette between puffs [4,5]. Particulate matter is an integral part of ETS [6]. PM_2.5_ is defined as a mixture of particles and droplets of 2.5 m in diameter or smaller, suspended in the air [7]. This fraction penetrates the smaller bronchi, bronchioles, and even the alveoli and therefore can exacerbate bronchial asthma [8,9].

Particulate matter (PM) is an independent risk factor for pulmonary and cardiovascular diseases. The smaller the particles are, the deeper they can penetrate into the lungs, and they may even enter into the blood stream. Studies have shown increasing morbidity and mortality in relation to PM exposure [10]. Li M. *et al.* [11] demonstrated the PM_2.5_-mediated induction of cellular processes that promote chronic obstructive pulmonary disease (COPD). These processes associated with an increase in COPD-hospitalizations and mortality [11].

However, smoking tobacco is not only one of the most popular legal drugs, but it also constitutes a high strain on the healthcare system because of the secondary diseases related to the consumption of tobacco products [12]. Lung cancer is probably the best-known secondary disease caused by smoking [13,14]. The lung is not the only part of the respiratory system that has direct contact with the toxic substances of tobacco smoke. The oral cavity is the first area to come into contact with inhaled tobacco smoke during the smoking process. Therefore, it is a potential hazard for the development of oral leukoplakia and cancer of the oral epithelium [15,16]. Carcinogenesis is the result of DNA-damage leading to an unregulated proliferation of cells [17]. Based on this theory, Cavallo *et al.* have found that direct DNA-damage induced by extracts from filter cigarettes is lower than that induced by unfiltered cigarettes [18]. SSS contains a significantly higher concentration of carcinogenic (>50) and toxic (>100) substances than MSS [4].

People in the environment near smokers are exposed to ETS that is associated with a high risk of lung cancer and other related health effects in non-smokers [19]. Third-hand smoke is also associated with decreased lung function and increased cancer risk [20]. It is defined as the residue of active smoking nicotine that lies on the surface of indoor environments (cars, clothes, curtains, wallpapers *etc.*) [21].

The risk of PM-associated morbidity and mortality is increased due to both active consumption of cigarettes and passive smoking. This associated risk is an approach to changing the classification of PM. Another way to categorize PM may be based on the hazard PM exposure poses to passive smokers, so we focus on the production of ETS in general, using a non-standard smoking machine instead of generating and analyzing mainstream smoke or side-stream smoke separately. Therefore, cigarettes were not conditioned prior to smoking.

## 2. Experimental Section

### 2.1. Cigarette Products

We investigated two different cigarette brands: the research cigarette 3R4F and two types of the brand Roth-Händle. 3R4F is a filter-tipped cigarette with a tar yield of 9.5 mg and a nicotine-yield of 0.73 mg per cigarette. Its total length is 84 mm, including a filter length of 27 mm. The filter-tipped Roth-Händle has the same total length as the reference cigarette and a filter length of 20 mm. The non-filter Roth-Händle has a size of circa (*ca.*) 80 mm. The Roth-Händle brand is produced by Reemtsma Cigarettenfabriken GmbH, which belongs to the Imperial Tobacco Group.

Tar and nicotine yield of all analyzed cigarettes are represented in Table 1. These parameters are based on the ISO 3308 smoking regime.

### 2.2. Automatic Environmental Tobacco Smoke Emitter (AETSE)

**Figure 1:**
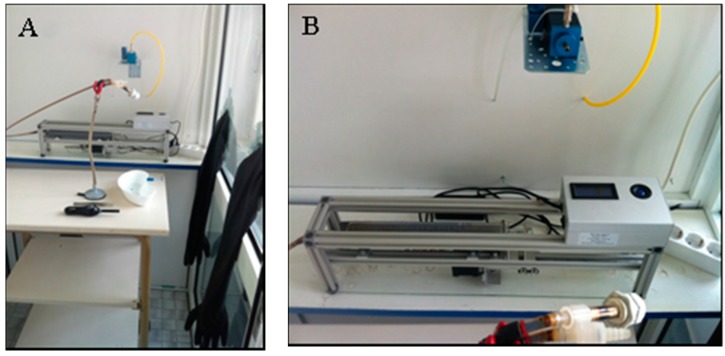
(**A**) The Automatic Environmental Tobacco Smoke Emitter (AETSE) in a glass chamber; (**B**) The AETSE in detail: a 200 mL glass syringe, a stepper motor, a microcontroller and stand equipment.

This study is part of the tobacco smoke particles and indoor air quality (ToPIQ) studies. The current Automatic Environmental Tobacco Smoke Emitter (AETSE) was steadily improved since its first use by Müller for the ToPIQ study protocol in 2011 [22]. A Norwegian engineering company (Schimpf-Ing, Trondheim, Norway) constructed the currently used smoking machine in 2013. The AETSE is illustrated in Figure 1A,B and it does not work according to the ISO 3308, unlike the Borgwardt RM20S^®^-syringe smoking machine [23].

A stepper motor pushes and pulls the syringe plunger of a 200-mL glass syringe, thus generating a negative pressure and sucking the MSS from the mouthpiece via pipes and two check valves into the ventilation chamber. The chosen smoking program was developed in house. Using this program and the AETSE, we tried to imitate human smoking behavior as realistically and reliably as possible within our financial and technical capabilities. The AETSE was programmed to automatically stop after 5 min of smoking. Meanwhile the side-stream smoke was emitted continuously from the smoldering cigarette.

**Figure 2:**
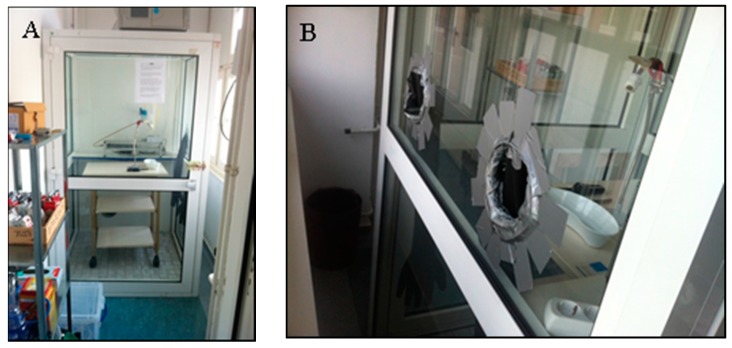
(**A**) The 2.88-m^3^ glass ventilation chamber in an air-conditioned laboratory room; (**B**) A pair of rubber gloves in the right wall of the chamber to reduce the potential of contact as much as possible.

The AETSE was put inside a 2.88-m^3^ glass ventilation chamber to protect laboratory staff from hazardous ETS. A pair of rubber gloves was inserted into the glass panel of the chamber’s right wall, permitting access to the AETSE without coming into contact to the toxic ETS emissions (Figure 2A,B).

**Figure 3:**
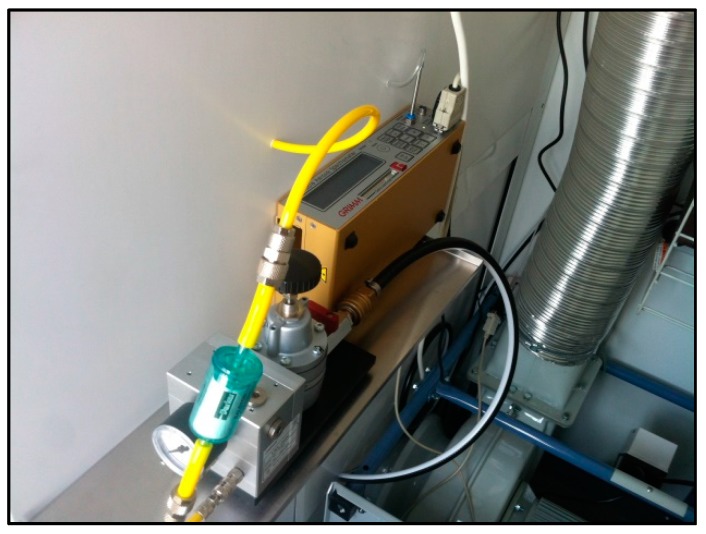
Aerosol spectrometer (yellow box) and pressure regulator with pressure indicator of the VKL mini. The dilution system VKL mini attenuates the sampled air in a ratio of 1:10 with neutral compressed air.

The aerosol spectrometer (Model 1.109, Grimm Co., Ainring, Germany) operates with a volume flow rate of 1.2 L/min and a sampling time of 6. Because the aerosol spectrometer is factory calibrated for environmental measurements in general but not specifically for tobacco smoke, measurement inaccuracies have to be assumed. To protect the sensitive measurement technology from the sticky, tar-containing condensates of ETS, a variable dilutor (“VKL-mini”, Model 7.951, Grimm Co., Ainring, Germany) was located at the backboard of the ventilation chamber to dilute the sampled air in a ratio of 1:10 with neutral compressed air (Figure 3).

The measurements were performed on three consecutive days. The temperature (22.5 ± 2 °C) and humidity (29% ± 5%) in the laboratory rooms were controlled by air conditioning. The background PM concentration was controlled at all times, whether measurements were taken or not.

### 2.3. The Smoking Protocol 

**Figure 4:**
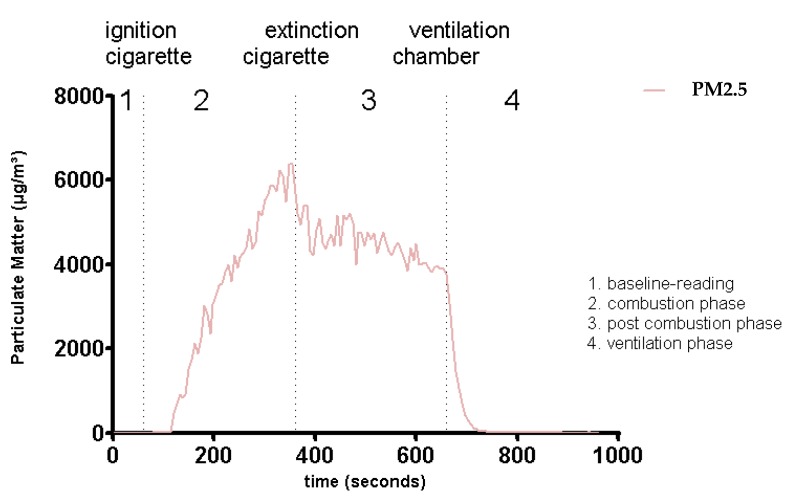
An example of a complete measuring cycle. Extinction not exctinction.

A self-developed smoking-protocol was used for this study. It does not pursue an international standardized smoking protocol. One measuring cycle is shown in Figure 4.

Each smoking cycle lasts 5 min and proceeds through a standardized sequence. First, a double puff is performed to sustain ignition of the cigarette, followed by an interval of 27 s until the next puff is initiated, each with a volume of 40 mL and a length of 3 s. Finally, the cigarette is dipped into a bowl of water to extinguish it. A complete measuring cycle lasts 16 min (Figure 4). It starts with a 1-min period of baseline reading, followed by a 5-min combustion phase (smoking cycle). Then, a post-combustion and a ventilation phase follow with a length of 5 min each. The chamber is ventilated at the end of each measuring cycle by high-performance industrial suction, located in the lower backboard and on the roof of the chamber. All PM is removed from the chamber and PM_2.5_ environmental baseline concentrations are achieved.

### 2.4. Data Processing and Data Analysis

**Figure 5:**
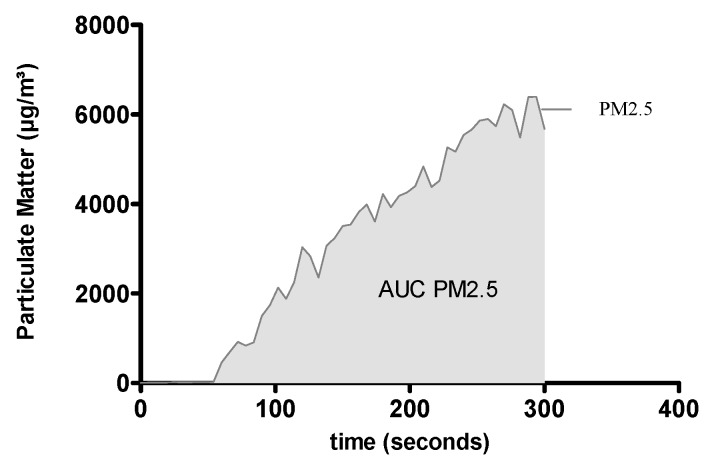
The interval from ignition to extinction (5 min) was evaluated by measuring the area under the curve (AUC) and particulate matter (PM).

Nineteen 3R4F reference cigarettes, filter-tipped Roth-Händle and non-filter Roth-Händle were smoked by the AETSE. We used the statistics program Graph Prism 5.03 (GraphPad Software, Inc., La Jolla, CA, USA) to analyze our collected data (Figure 5).

**Figure 6:**
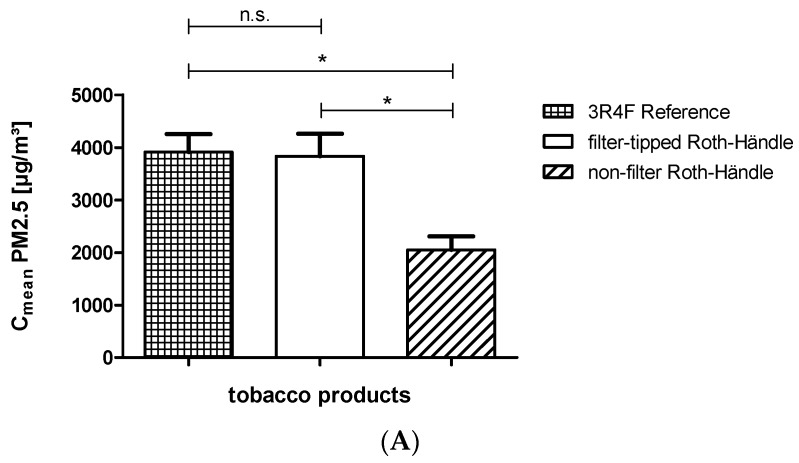

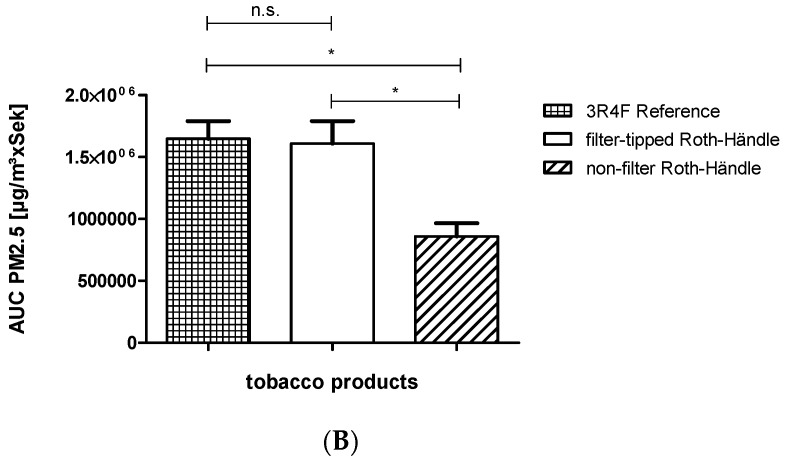
Comparison of the 3R4F, filter-tipped Roth-Händle and non-filter Roth-Händle. Significance was assumed when *p* < 0.05 (* means significant). (**A**) PM_2.5_ mean concentrations and (**B**) PM_2.5_ AUC are compared.

Only PM_2.5_ was used for calculations. Both mean concentration (C_mean_) and area under the curve (AUC) were calculated for each cigarette, and data were charted in column diagrams including standard deviation (Figure 6A,B). 

**Figure 7:**
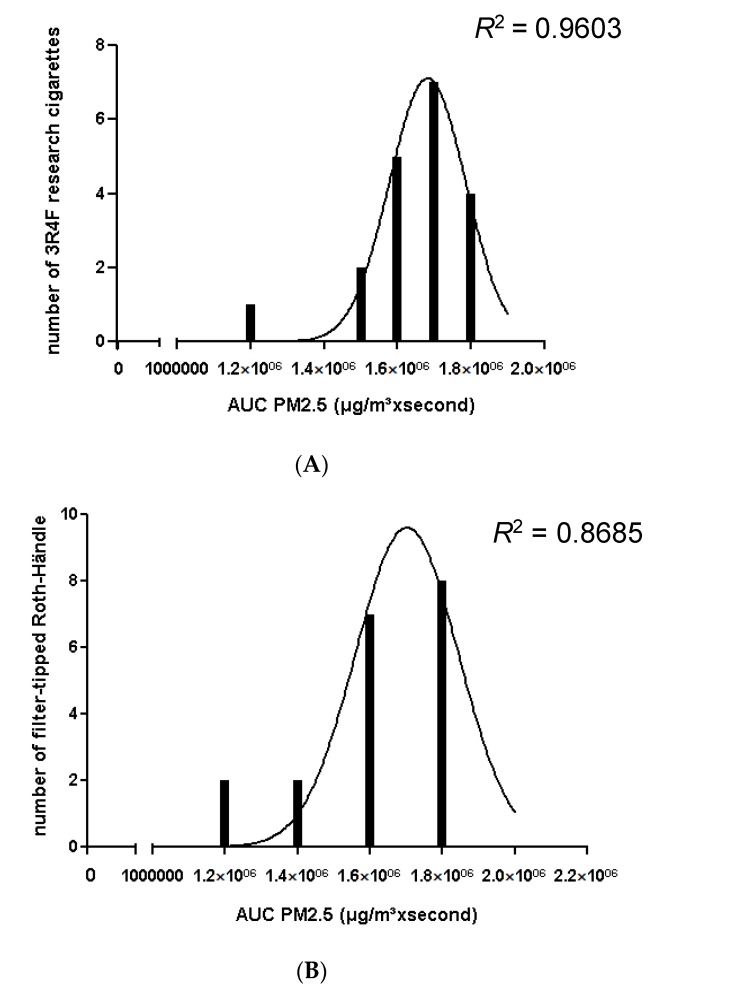

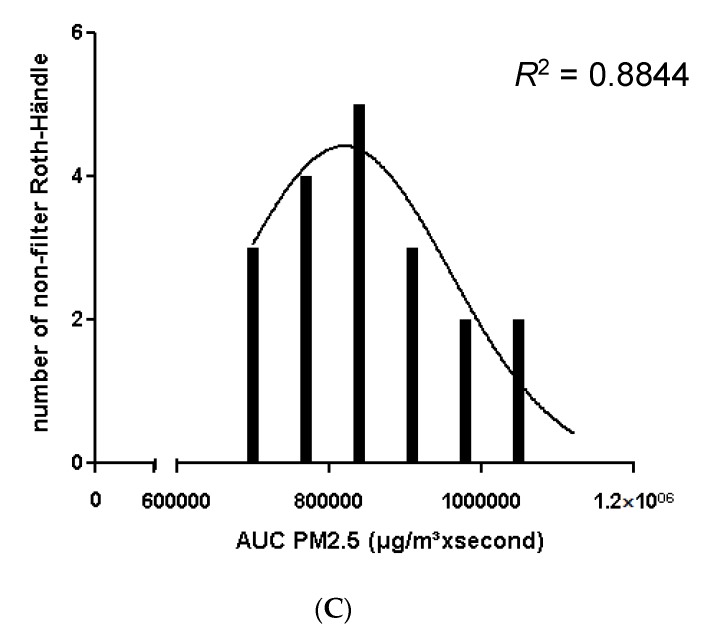
(**A**) Bar chart of AUC against the number of cigarettes with Gaussian curve for the 3R4F reference cigarettes; (**B**) filter-tipped Roth-Händle and (**C**) non-filter Roth-Händle.

Neither filter ventilation nor total ventilation was taken into account in the data processing and analysis. After testing individual parameters for Gaussian distributions (Figure 7A–C), one sample *t*-test was performed to show significant differences. Bonferroni’s correction was not necessary because we only compared two kinds of cigarettes in every case. Significant differences of AUC and C_mean_ for PM_2.5_ between 3R4F reference cigarettes, filter-tipped Roth-Händle and non-filter Roth-Händle cigarettes were assumed when *p* < 0.05.

## 3. Results and Discussion

### 3.1. Results

PM_2.5_ mean concentration and PM_2.5_ AUC of all investigated cigarettes proved to be normally distributed (Figure 7A–C). The one sample *t*-test showed significant differences for the 3R4F reference cigarette *versus (vs.)* the non-filter Roth-Händle and the filter-tipped Roth-Händle *vs.* the non-filter Roth-Händle (Figure 6). As mentioned previously, significance was assumed when *p* < 0.05.

We found that the data are nearly twice as high as the same parameters for the non-filter Roth-Händle for mean concentration and AUC for the 3R4F reference cigarette and for the filter-tipped Roth-Händle. Detailed results of our research are presented in Table 2.

### 3.2. Discussion

Our measurements demonstrated that cigarettes with ventilated filters release more side-stream smoke for the combustion of a given mass of tobacco than do non-filter cigarettes, so our data corresponded with our findings in 2015 [6] and with the findings of Thapliyal *et al.* in 2004 [24]. Thapliyal *et al.* [24] found similar results by comparing Indian non-filter cigarettes with Indian filter King cigarettes using the international standard smoking regime ISO 3308.

We found significant differences (*p* < 0.05) between the 3R4F reference cigarette/filter-tipped Roth-Händle and non-filter Roth-Händle. Because of that, our null hypothesis (H0) that declares no difference between the researched cigarettes could be rejected. The comparison between different brands showed that the amount of produced PM does not significantly depend on the brand, but, among other things, also on the presence of a filter. We could not find a significant difference for mean concentration and AUC of PM_2.5_ between the 3R4F reference cigarette and the filter-tipped Roth-Händle. 

Moreover, it is important to note that our data are relative values that reflect significant differences concerning the mean concentration of PM_2.5_ in the emitted ETS between filter-tipped and non-filter cigarettes. The aerosol spectrometer is not just calibrated on tobacco smoke, as in Dacunto *et al.* [25]. This method underlines that the calibration of the measuring gadget does not correspond significantly with the determined data. We achieved our basic objective of showing a significant difference in emitted PM_2.5_ between the researched cigarettes with a relatively simple aerosol spectrometer that does not underlie a standard smoking regime according to ISO 3308. However, we also know that it is necessary to generate a correction factor for ETS in terms of a gravimetric filter to ascertain absolute measured data concerning ETS in further studies.

The presence of a cigarette filter may be one aspect of increased ETS due to the 3R4F reference cigarette and filter-tipped Roth-Händle as compared with the non-filter Roth-Händle. The filter elevates the resistance in the cigarette [26], reducing the oxygen level to induce a high-temperature combustion compared to non-filter cigarettes. This results in an incomplete combustion of the tobacco in filter-tipped cigarettes. Additionally, filter ventilation amplifies the combustion. Moreover, the volume and velocity of the air moving through the shaft of the filter-ventilated cigarette was decreased because of the perforations in the cigarette filter [27]. This also leads to an increased amount of ETS and decreased combustion [28]. These explanations underline, on the one hand, the relevance of a cigarette filter, and on the other hand, the presence of filter ventilation because of perforations. However, in the case of our study, neither filter ventilation nor total ventilation was taken into account. Further studies could include cigarettes without filter ventilations to make the results even more comparable. 

Furthermore, the higher mean concentration of PM_2.5_ in the ETS of the filter-tipped Roth-Händle and the 3R4F reference cigarette is the result of the functional principle of the processed filter. The functionality of the cigarette filter can be compared with a fine particulate air filter of the type used in cars or factories: both are constructed to protect the environment from particulate matter [29]. However, in the case of cigarettes, the filter will primarily reduce the toxic substances in the inhaled smoke and thus smoking-associated diseases [30]; however, passive smokers are still inadequately protected.

The insufficient protection of people in the immediate environment of smokers is also enhanced by the different streams of the ETS. Emitted smoke can be divided into mainstream and side-stream smoke; side-stream-smoke comprises approximately 85% of the ETS [17]. It is produced from the cigarette smoldering between the drags of the smoker [18] and is not influenced by a cigarette filter. The hazard due to passive smoking has also been shown in investigations in 1987 and 2005: side-stream is more toxic than mainstream smoke [31,32].

In our opinion, the concentration of particulate matter should be considered with regard to tobacco tax. We already detected significantly higher amounts of PM_2.5_ in the smoke of brand cigarillos compared to cigarettes of the same brand [3,33]. Currently, taxation of tobacco products in Germany differs between cigarettes, fine-cut rolling tobacco, pipe tobacco, cigars and cigarillos [34]. Consequently cigarillos are taxed less than, for example, cigarettes, even though they emit distinctly more PM_2.5_ [33]. A PM-dependent taxation could be another effective way to protect people from active and passive smoking; higher amounts of ETS emitted by the tobacco product should result in an increase in tobacco tax for that product. This may cause low-income smokers, who spend much of their time and money on tobacco use, to reduce their tobacco consumption [35]. Guillamier *et al.* demonstrated the priority of cigarettes for Australian socioeconomically disadvantaged smokers despite rising cigarette prices. Instead of reducing or quitting smoking, the smokers studied reduced spending on things like food or opted to pay their bills late [36]. A cigarette tax and price increase in Korea lead to a twofold greater reduction of tobacco consumption in the lowest income quartile than among smokers in the top income quartile [37].

## 4. Conclusions

It is uncontroversial that smoking has a relevant but also avoidable effect on our health and health care system because of smoking-associated diseases. Both exhaled tobacco smoke and side-stream smoke contain huge amounts of PM_2.5_, which is responsible for the development and chronification of airway diseases (e.g., COPD or bronchial asthma). We have been able to show that whether a cigarette includes a filter seems to have a much higher impact on the amount of ETS-associated PM_2.5_ emitted by the tobacco product than does the brand of cigarette. Meanwhile, a filter-tipped cigarette is believed to be a less harmful choice because of the reduced toxicity of the inhaled MSS for smokers. In light of our findings, efforts to protect non-smokers, especially children, from ETS exposure in private households and cars seem to be underdeveloped in most countries. This led Great Britain to pass legislation on 1 October 2015, that bans smoking in private cars when underage children are passengers. 

The only efficient way to protect people from the harmful effects of active and passive smoking is to significantly reduce tobacco consumption in society. It is extremely difficult to protect nonsmokers by law in private environments; however, smokers may limit smoking in response to taxation increases on cigarettes and other tobacco products.

## Figures and Tables

**Table 1:** Tar yield and nicotine yield for the 3R4F reference cigarettes, filter-tipped Roth-Händle and non-filter Roth-Händle.

Tobacco Products	Tar Yield Per Cigarette (mg)	Nicotine Yield Per Cigarette (mg)
3R4F reference	9.5	0.73
Filter-tipped Roth-Händle	10	0.7
Non-filter Roth-Händle	10	1.0

**Table 2:** Shows average PM_2.5_ mean concentrations and average AUC PM_2.5_ for the 3R4F reference cigarettes, filter-tipped Roth-Händle and non-filter Roth-Händle.

Tobacco Products	C_mean_PM_2.5_ (μg/m^3^)	AUC PM_2.5_ (μg/m^3^·s)	Combustion Time (s)
3R4F reference	3911 ± 341.1	1,647,006 ± 144,273	300
Filter-tipped Roth-Händle	3831 ± 429.2	1,608,000 ± 181,736	300
Non-filter Roth-Händle	2053 ± 254.7	858,891 ± 107,655	300

## References

[1] Mund M., Kloft B., Bundschuh M., Klingelhoefer D., Groneberg D.A., Gerber A. (2014). Global research on smoking and pregnancy—A scientometric and gender analysis. Int. J. Environ. Res. Public Health.

[2] Mund M., Louwen F., Klingelhoefer D., Gerber A. (2013). Smoking and pregnancy—A review on the first major environmental risk factor of the unborn. Int. J. Environ. Res. Public Health.

[3] Gerber A., Bigelow A., Schulze M., Groneberg D.A. (2015). Brand cigarillos—A cheap and less harmful alternative to cigarettes? Particulate matter emissions suggest otherwise. Int. J. Environ. Res. Public Health.

[4] Singer M.V., Batra A., Mann K. (2010). Alkohol und Tabak: Grundlagen und Folgeerkrankungen.

[5] Wong L.S., Martins-Green M. (2004). Firsthand cigarette smoke alters fibroblast migration and survival: Implications for impaired healing. Wound Repair Regen..

[6] Gerber A., Hofen-Hohloch A.V., Schulze J., Groneberg D.A. (2015). Tobacco smoke particles and indoor air quality (ToPIQ-II)—A modified study protocol and first results. J. Occup. Med. Toxicol..

[7] Neininger S. (2010). Die Entstehung von Feinstaub und die Gefahren für Mensch und Umwelt.

[8] Harrison R.M., Yin J. (2000). Particulate matter in the atmosphere: which particle properties are important for its effects on health?. Sci. Total Environ..

[9] Balmes J.R., Cisternas M., Quinlan P.J., Trupin L., Lurmann F.W., Katz P.P., Blanc P.D. (2014). Annual average ambient particulate matter exposure estimates, measured home particulate matter, and hair nicotine are associated with respiratory outcomes in adults with asthma. Environ. Res..

[10] Anderson J.O., Thundiyil J.G., Stolbach A. (2012). Clearing the air: A review of the effects of particulate matter air pollution on human health. J. Med. Toxicol..

[11] Li M.-H., Fan L.-C., Mao B., Yang J.-W., Choi A.M.K., Cao W.-J., Xu J.-F. (2015). Short term exposure to Ambient fine particulate matter (PM_2.5_) increases hospitalizations and mortality of chronic obstructive pulmonary disease: A systematic review and meta-analysis. CHEST J..

[12] Gawlik F.M. (2009). Möglichkeiten der Raucherprävention bei Jugendlichen: Pilotstudie zu einem Raucherpräventionsprogramm der Universitätsklinik Freiburg und dessen Auswirkungen auf das Verhalten von Schülern.

[13] Ito H., Matsuo K., Tanaka H., Koestler D.C., Ombao H., Fulton J., Shibata A., Fujita M., Sugiyama H., Soda M. (2011). Nonfilter and filter cigarette consumption and the incidence of lung cancer by histological type in Japan and the United States: Analysis of 30-year data from population-based cancer registries. Int. J. Cancer.

[14] Brownson R.C., Eriksen M.P., Davis R.M., Warner K.E. (1997). Environmental tobacco smoke:Health effects and policies to reduce exposure. Annu. Rev. Public Health.

[15] Fu J.Y., Gao J., Zhang Z.Y., Zheng J.W., Zhong L.P., Luo J.F., Xiang Y.B. (2013). Role of cigarette filter on the risk of oral cancer: A case-control study in a Chinese population. Oral Dis..

[16] Macigo F.G., Mwaniki D.L., Guthua S.W., Njeru1 E.K. (2001). Influence of cigarette filters on the risk of developing oral leukoplakia in a Kenyan population. Oral Dis..

[17] Böcker W., Aguzzi A. (2008). Pathologie.

[18] Cavallo D., Ursini C.L., Fresegna A.M., Maiello R., Ciervo A., Ferrante R., Buresti G., Iavicoli S. (2013). Cyto-genotoxic effects of smoke from commercial filter and non-filter cigarettes on human bronchial and pulmonary cells. Mutat. Res..

[19] Behera S.N., Xian H., Balasubramanian R. (2014). Human health risk associated with exposure to toxic elements in mainstream and sidestream cigarette smoke. Sci. Total Environ..

[20] Ramírez N., Özel M.Z., Lewis A.C., Marcé R.M., Borrull F., Hamilton J.F. (2014). Exposure to nitrosamines in thirdhand tobacco smoke increases cancer risk in non-smokers. Environ. Int..

[21] Giraldi G., de Ruggiero G.F., Marsella L.T., de Luca d’Alessandro E. (2013). Environmental tobacco smoke: Health policy and focus on Italian legislation. Clin. Ther..

[22] Mueller D., Uibel S., Braun M., Klingelhoefer D., Takemura M., Groneberg D.A. (2011). Tobacco smoke particles and indoor air quality (ToPIQ)—The protocol of a new study. J. Occup. Med. Toxicol..

[23] Garcia-Canton C., Errington G., Anadon A., Meredith C. (2014). Characterisation of an aerosol exposure system to evaluate the genotoxicity of whole mainstream cigarette smoke using the *in vitro* γH2AX assay by high content screening. BMC Pharmacol. Toxicol..

[24] Thapliyal R., Dolas S.S., Pakhale S.S., Maru G.B. (2004). Evaluation of DNA damage in mice topically exposed to total particulate matter from mainstream and sidestream smoke from cigarettes and bidis. Mutagenesis.

[25] Dacunto P.J., Cheng K.-C., Acevedo-Bolton V., Jiang R.-T., Klepeis N.E., Repace J.L., Ott W.R., Hildemann L.M. (2013). Real-time particle monitor calibration factors and PM_2.5_ emission factors for multiple indoor sources. Environ. Sci..

[26] Dittrich D.J., Fieblekorn R.T., Bevan M.J., Rushforth D., Murphy J.J., Ashley M., McAdam K.G., Liu C., Proctor C.J. (2014). Approaches for the design of reduced toxicant emission cigarettes. Springerplus.

[27] Harris B. (2011). The intractable cigarette “filter problem”. Tob. Control.

[28] Siu M., Mladjenovic N., Soo E. (2013). The analysis of mainstream smoke emissions of Canadian “super slim” cigarettes. Tob. Control.

[29] Fiebig M., Wiartalla A., Holderbaum B., Kiesow S. (2014). Particulate emissions from diesel engines: Correlation between engine technology and emissions. J. Occup. Med. Toxicol..

[30] Crooks I., Scott K., Dalrymple A., Dillon D., Meredith C. (2015). The combination of two novel tobacco blends and filter technologies to reduce the *in vitro* genotoxicity and cytotoxicity of prototype cigarettes. Regul. Toxicol. Pharmacol..

[31] Adams J.D., O’Mara-Adams K.J., Hoffmann D. (1987). Toxic and carcinogenic agents in undiluted mainstream smoke and sidestream smoke of different types of cigarettes. Carcinogenesis.

[32] Schick S., Glantz S. (2005). Philip Morris toxicological experiments with fresh sidestream smoke: More toxic than mainstream smoke. Tob. Control.

[33] Wasel J., Boll M., Schulze M., Mueller D., Bundschuh M., Groneberg D.A., Gerber A. (2015). Brand cigarillos: Low price but high particulate matter levels—Is their favorable taxation in the European Union justified?. Int. J. Environ. Res. Public Health.

[34] Gesetze im Internet. http://www.gesetze-im-internet.de/tabstg_2009/__2.html.

[35] Chaloupka F.J., Straif K., Leon M.E. (2011). Effectiveness of tax and price policies in tobacco control. Tob. Control.

[36] Guillaumier A., Bonevski B., Paul C. (2015). “Cigarettes are priority”: A qualitative study of how Australian socioeconomically disadvantaged smokers respond to rising cigarette prices. Health Educ. Res..

[37] Choi S.E. (2014). Are lower income smokers more price sensitive?: The evidence from Korean cigarette tax increases. Tob. Control.

